# A strategy analysis for genetic association studies with known inbreeding

**DOI:** 10.1186/1471-2156-12-63

**Published:** 2011-07-18

**Authors:** Stefano Cabras, Maria Eugenia Castellanos, Ginevra Biino, Ivana Persico, Alessandro Sassu, Laura Casula, Stefano del Giacco, Francesco Bertolino, Mario Pirastu, Nicola Pirastu

**Affiliations:** 1 Department of Mathematics and Informatics, University of Cagliari, Cagliari, Italy; 2 Department of Statistics and O. R., Rey Juan Carlos University, Mostoles, Madrid, Spain; 3 Institute of Population Genetics CNR, Alghero (SS), Italy; 4 Shardna Live Science, Loc. Piscina Manna, Pula (CA), Italy; 5 Institute of Molecular Genetics CNR, Pavia, Italy; 6 Department of Medical Science, University of Cagliari, Cagliari, Italy; 7 Medical Genetics, IRCCS-Burlo Garofolo, University of Trieste, Trieste, Italy

## Abstract

**Background:**

Association studies consist in identifying the genetic variants which are related to a specific disease through the use of statistical multiple hypothesis testing or segregation analysis in pedigrees. This type of studies has been very successful in the case of Mendelian monogenic disorders while it has been less successful in identifying genetic variants related to complex diseases where the insurgence depends on the interactions between different genes and the environment. The current technology allows to genotype more than a million of markers and this number has been rapidly increasing in the last years with the imputation based on templates sets and whole genome sequencing. This type of data introduces a great amount of noise in the statistical analysis and usually requires a great number of samples. Current methods seldom take into account gene-gene and gene-environment interactions which are fundamental especially in complex diseases. In this paper we propose to use a non-parametric additive model to detect the genetic variants related to diseases which accounts for interactions of unknown order. Although this is not new to the current literature, we show that in an isolated population, where the most related subjects share also most of their genetic code, the use of additive models may be improved if the available genealogical tree is taken into account. Specifically, we form a sample of cases and controls with the highest inbreeding by means of the Hungarian method, and estimate the set of genes/environmental variables, associated with the disease, by means of Random Forest.

**Results:**

We have evidence, from statistical theory, simulations and two applications, that we build a suitable procedure to eliminate stratification between cases and controls and that it also has enough precision in identifying genetic variants responsible for a disease. This procedure has been successfully used for the beta-thalassemia, which is a well known Mendelian disease, and also to the common asthma where we have identified candidate genes that underlie to the susceptibility of the asthma. Some of such candidate genes have been also found related to common asthma in the current literature.

**Conclusions:**

The data analysis approach, based on selecting the most related cases and controls along with the Random Forest model, is a powerful tool for detecting genetic variants associated to a disease in isolated populations. Moreover, this method provides also a prediction model that has accuracy in estimating the unknown disease status and that can be generally used to build kit tests for a wide class of Mendelian diseases.

## Background

One of the main objectives in studying the genetics of complex diseases is not only the search of genetic variants associated to pathologies [1], but also to build predictive models which help both their diagnosis and early treatment. This problem can be formalized by expressing the disease status, *Y*, of each subject as a Bernoulli random variable *Y *= {0, 1} where *Y *= 1 indicates an affected subject. The main quantity of interest, *F*(**x**) = Pr(*Y *= 1|**x**), is the conditional probability of being affected given a set **x **of genetic variants and environmental variables. Such variables form a huge set of potential predictors, which we will refer to as *omic profile*. Essentially, , where *P *is the number of considered genetic variants and environmental variables. Specifically, we use a sample of *N *≪ *P *where *N *is of order of hundreds while *P *is thousands times larger. This setup complicates the estimation of *F*(**x**), because, in absence of strong prior information [2] on the part of the omic profile related to the disease, the data should allow us to choose *F*(**x**) within a large class of models . In order to achieve this goal it is necessary to reduce the spurious genetic variability not related to *Y*. For example, if we were using logistic regression models, *F*(**x**) would be one of these models selected between all the possible logistic regression models, , which are, at least, 2*^P^*. In this paper we relax the assumption of a parametric model by using non-parametric methods [3], which means that  has infinite dimension. Such models are usually referred as non-parametric models. The genomic profile, part of the omic profile, consists of a large set of DNA markers, say 500000 Single-Nucleotide Polymorphisms (SNPs), while the set of environmental variables includes individual anthropometric measurements and information derived from a standardized interview collecting socio-demographic, lifestyle, medical and pharmacological history data on many pathologies. We suppose that such covariates may cause the outcome *Y*. In particular, for certain types of diseases, it is possible to have prior information about the environmental variables, but in most cases there is no such information about the causing genes. The disease prediction model, *F*(**x**), for the future outcome *Y *|**x **must take into account gene-gene interactions and also their interaction with the environment. Such interactions, usually of unknown order, can be multiplicative or additive [4]. Estimation of *F *(**x**) is a primary concern in personalized medicine, because *F*(**x**) can be used as the basis for early diagnosis of a disease, permitting actions to prevent the pathology before its insurgence, and to personalize treatments.

In order to estimate *F*(**x**), we consider a matrix of omic profiles, **X***_N _*_× *P*_, and the known disease status, **y***_N _*= (*y*_1_, *y*_2_, ..., *y_N _*), measured on *N *≈ 100 highly inbred individuals that belong to an isolate population where the genealogy is fully known.

The translation of this estimation problem into statistical terms sounds as follows: given a huge set of covariates, *P *≈ 500000, we have to estimate a probability model, , using a sample of dependent observations, (**y***_N _*, **X***_N _*_× *P *_), of size *N *≪ *P*. The statistical analysis of such problem presents the following critical points:

*i*) observations are not independent and consequently all unconditional inference, with respect to the genealogy, cannot be applied here. Differently from usual association studies between genetic variants and diseases, we have knowledge of such dependency by means of the genealogical tree. Moreover, the dependency is important in order to gain precision in estimating *F*(**x**). In fact, two affected brothers are more likely to share the same part of **x **causing the disease with respect to two unrelated subjects.

*ii*) the estimation of model *F*(**x**) would typically lead to a sparse model because biological background suggests that only a very small set of genetic variants interact in order to produce the disease. The dimension of  grows exponentially with *P*. For example, if SNPs configurations were represented by categorical variables with two levels the space  would have dimension 2*^P^*, without considering interactions. Such dimension prevents an exhaustive exploration of all possible models. Moreover, as *N *≪ *P *then classical multivariate analysis techniques, such as multivariate regression, cannot be employed here to make an exhaustive search of all possible models. Finally, usual model selection approaches are not feasible due to computational costs.

In this paper, we aim to address the above critical points. In particular, point *i*) is considered in the Methods Section by reducing the genetic variability not related with the disease. We achieve this through an experimental design in which we choose, for each case, the most related control, based on the known genealogy. Point *ii*) is treated also in the Methods Section where Random Forest, a non-parametric regression model based on ensemble methods, is employed to estimate *F*(**x**). This allows us to explore a wide region of  at the price of reasonable computational costs.

For validation purposes, we present applications of the method to two different phenotypes: Beta-Thalassemia and common asthma. Beta-Thalassemia is a genetic disorder caused by a mutation inside the beta-hemoglobin (HBB) gene [5]. Only homozygous individuals for the mutation manifest the clinical traits of the disease. However, carriers, although completely sane, show a reduced mean cell volume (MCV≤72) of red blood cells [6], and this parameter can be used to identify them. In Sardinia carrier of beta-thalassaemia are about 15% of the population and a single mutation account for 95% of the beta-thalassaemia mutations [7,8]. The main goal of this analysis is to validate the method by tracing back the position of the mutation in the gene.

Differently from Beta-thalassemia, the goal of analyzing common asthma is to gain more biological insights on this diffuse disease which may be caused by several unknown variants on different genes.

Although the method here proposed is of general applicability to any isolated population, we tested it on a population located in one small village (Talana) in a secluded area (Ogliastra) of Sardinia (Italy). Such population is characterized by a great deal of homogeneity in life style and eating habits and by a high endogamy and consanguinity. Inhabitants of the village participated to an epidemiological survey assessing their health status, so that a complete and standardized data set is available. Thanks to the accessibility of complete municipal and parish archives, going back to the seventeenth century, it was possible to cluster all people living in the villages into large familiar structures with common ancestor. Data have been collected by Shardna Live Science http://www.shardna.com within the Ogliastra - project aimed at studying several genetic isolates of Ogliastra.

## Results and Discussion

The following two sections present the application of the method here proposed to beta-Thalassemia and to common asthma.

### Application to Beta-Thalassemia

One of the biggest issue in genome wide association studies is the number of false positive results due to the great number of association tests performed. In case-control studies it should be possible to reduce such number by choosing the control subjects so that they are the most genetically similar to the cases. We propose to apply the Hungarian algorithm [9,10] to solve this problem by using the known kinship coefficient [11] as a measure of the genetic similarity between the subjects. The proposed approach also works if the kinship were estimated from the sample, which is however not the case here. The details on how the choice of the controls is done are described in the method section.

The aim of this application is to evaluate the power of the Hungarian method in choosing the best set of controls for a given sample of cases which correspond to all the Beta-Thalassemia mutation carriers in the isolated population we investigated. Such cases can be easily identified, among the available data, through the use of the MCV values measured for each subject in our study. In fact Beta-Thalassemia mutation carriers are known to show reduced MCV, namely we used MCV≤72 for cases and MCV≥75 for controls. With such cutoffs there are 123 cases, over a data set of 805 subjects. We then selected the subsample **D**, where 123 controls have been chosen to be the most related to the *I *= 123 cases, based on the kinship coefficient matrix of the 805 subjects. Analysis of such data is compared against other 9 balanced subsamples, of size *N *= 246, where controls have been assigned randomly from the available 682 controls. Such assignation, that ignore the relativeness, is the usual one for observational studies from outbred populations.

**Figure 1:**
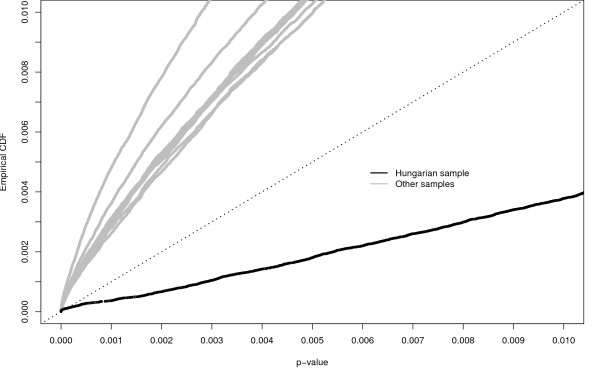
**Empirical distributions of the *p*-values for Thalassemia's analysis**. Empirical distributions of the *p*-values of Fisher test based on **D **and on other balanced subsamples where controls have been assigned randomly to cases.

Usual association studies are of single-point type and make use of hypothesis testing to evaluate the association between **y***_N _*and each SNP [12]. In order to evaluate if our subsample **D **would potentially provide a less number of false positives, due to the elimination of population stratification effect, we performed a genome wide scan on each of the 10 samples using Fisher exact test, that is, for each sample, we evaluate the evidence of the hypothesis of independence between *Y *and each one of *P *= 429378 SNPs. All tests are compared by means of their corresponding *p*-values. Figure 1 reports the most interesting part of the empirical cumulative distribution function (cdf) of the *p*-values less than 0.01 for each one of the ten considered subsamples. Beta-Thalassemia is a monogenic disease and therefore we expect a small proportion of SNPs associated with the *Y *status. We can see that, when using **D**, the proportion of p-values below 0.001 is smaller than the corresponding ones obtained with the other subsamples. This provides the first evidence of the reduction of the genetic variability operated by the introduction of genealogical information by means of the Hungarian method. We note that the rank of SNPs produced by the very simple Fisher test, used in the simulation study for Thalassemia, is the same as the one produced by the Benjamini Hochberg (BH) method discussed in [13]. This is because ordered *p*-values are multiplied for the increasing sequence *i*/*P*, *i *= 1, ..., *P*. As the BH method controls the False Discovery Rate (FDR) under the condition of positive dependence among tests discussed in [14], this implies that the rank of SNPs obtained for the Thalassemia is the same as those that we would have obtained also considering the dependency among tests. The analysis here performed can be also viewed as a kind of multi-point analysis. We further pursue this comparison by looking at the prediction errors, Λ, of the estimated Random Forest  model, , based only on the most important SNP and its distance from the HBB gene. We have one important SNP for each data set and this SNP has been selected using the Bagging approach as detailed in the Methods Section. The Bagging approach is enough in this application because the Beta-Thalassemia is a monogenic disease. Prediction errors and distances, for all samples, are reported in Table 1. We can see that only using sample **D**, the prediction error is the lowest (12%) and the selected SNP is also one of the nearest SNP to the HBB gene. Using other samples, we obtain larger prediction errors that often point to SNPs that are farther from the HBB gene.

**Table 1:** Comparison of prediction errors for Thalassemia's analysis

**Sample Id**.	First SNPs	Error (%)	Distance (in Kb)
*Hungarian*	rs7124435	12	394
Random 1	rs12271916	32	581
Random 2	rs12271916	32	581
Random 3	rs16932946	33	278
Random 4	rs12271916	32	581
Random 5	rs12271916	32	581
Random 6	rs12271916	32	581
Random 7	rs1378738	32	316
Random 8	rs12271916	32	581
Random 9	rs12271916	32	581

Prediction error and distance from the HBB gene for the first important SNP for all analyzed samples in Thalassemia's analysis.

Comparing  approach with the single-point analysis, carried out with Fisher test on subsample **D**, we obtain that SNP rs12271916 is the most significantly associated according to the Fisher test, but it is also located at 580 *Kb *from the HBB gene farther away from the SNP obtained with .

The final message conveyed by Figure 1 and Table 1 is that the subsample **D **has lower genetic spurious variability than other subsamples obtained ignoring the genealogy. Finally, using  with the genealogical tree we are more likely to trace back the position of the mutation point causing Beta-Thalassemia.

### Application to Asthma

Over a data set of 208 genotyped subjects underwent clinical and instrumental examination, we dispose of a total of *I *= 57 asthmatic cases and *J *= 151 non asthmatic potential controls. For each subject we measured breath nitric oxide, forced expiratory volume (FEV1) and blood IgE levels. Moreover a standardized epidemiological questionnaire was administered to all participants to the study. The affection status has been then assessed by a clinician according to the GINA (Global INitiative for Asthma) guidelines. The total amount of considered SNPs is *P *= 500192, each of them with two or three configurations. For each individual sex, age, smoking habits and degree of physical and sport activity is known. The latter has been classified in three categories: scarce, moderate and intense. We also dispose of 561 further subjects who did not undergo a clinical assessment for asthma but did participate to the epidemiological survey reporting their health status for whom genotypes are available. This sample will be used for validation purposes by looking at the association of the asthma status, predicted by the  method, with asthma status self-reported by subjects.

**Figure 2:**
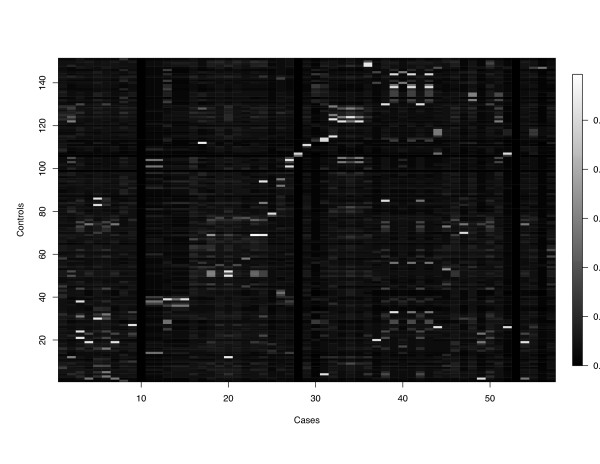
**Kinship matrix for Asthma's analysis**. Kinship matrix between cases and controls. Brightest rectangles define controls that are paired with cases.

The genealogical tree has been summarized into the matrix of kinship coefficients, K*, showed in Figure 2. High coefficients are symbolized by bright rectangles that also indicate highly related individuals which constitute the subsample, **D**, used to estimate .

After a first screening with the Bagging approach, from *P *variables, we end up with a list of *P*' = 200 variables. Such variables are all SNPs, while environmental variables do not play almost any role in predicting asthma if compared with these 200 SNPs.

**Figure 3:**
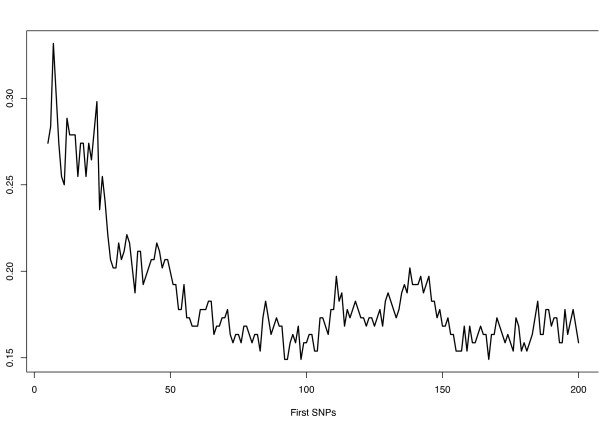
**Out Of Bag prediction errors for Asthma's analysis**. Out Of Bag prediction errors of  with the first 200 most important SNPs.

From Figure 3 we can see how the estimated prediction error, Λ, decreases when using  with the first most important SNPs, in particular with the first 100 SNPs, used for the final model, the estimated value of Λ is about 15%. It is important to stress that 15% is almost three times less than 50%, which is the initial classification error of a balanced sample.

We further validate the estimated prediction model , with the first 100 most important SNPs, on the 561 genotyped individuals, not including in the previous *I *+ *J *= 208 subjects, by looking at the association of the predicted asthma state, , with the self-reported diagnosis. It is worth noting that comparing the clinically assessed individuals to the self-reporting ones we observed an agreement on the 90.9% of subjects. The association between  and the anamnestic data is highly significative (*p*-value ≈ 10^-6 ^). Among the 68 asthmatic, 52 have been correctly classified by , while the 493 non-asthmatic 271 have been correctly classified. Essentially we have that individuals predicted to be healthy and having an healthy anamnesi are more than the number expected under the hypothesis of independence between anamnesi and the predicted asthma status, . The same, but in the reverse, can be noted for the frequency of those classified as asthmatic which also exhibit an asthmatic anamnesi more than that expected under the hypothesis of independence.

This evidence may suggest that the 100 SNPs, which we claim to be important in classification, are functionally related to the biological processes underling common asthma. However, these results are speculatory and would need further validation. The first ten most important SNPs found with  are reported in Table 2. The most important SNP is inside *P KNOX*2, a transcription factor which has been shown to be up-regulated in vascular endothelial cells stimulated by interleukin 4 [15], which is one of the most replicated gene in asthma [16]. This gene could act on asthma downstream the IL4 taking effect on the insurgence of the pathology. The second SNP is inside Hs.462615, for which almost no information is available, except that it is a sequence found expressed in pulmonary artery endothelial cells (human UniGene). The third most important SNP is inside gene ENST00000261401 that codifies the Coronin of type 1. Coronin of type 1 binds the actin protein which has been associated to non allergic asthma in rats according to [17]. We further applied the Mixed Effect (ME) model discussed in [18-20] to all 208 available subjects and we end up with a list of the 10 most important SNPs (Table 3) which differ from those obtained with the  as they are located on different genomic regions. The main problem with ME model is that we cannot regress the binomial response *Y *against all *P *SNPs, but instead we have to regress each SNP separately, thus losing interactions that may exists between SNPs.

**Table 2:** Asthma's analysis with  model

Order	SNPs	Gene	**Chrom**.	**Chrom. Pos**.
1	rs12273350	PKNOX2	11	125241046
2	rs1254673	no	10	44473793
3	rs10861957	CORO1C	12	109056377
4	rs3105377	no	7	68837002
5	rs10004892	no	4	189872850
6	rs434949	no	11	29602274
7	rs9524111	GPC6	13	94169901
8	rs1918215	no	12	77750550
9	rs7958647	no	12	77747825
10	rs10746129	CORO1C	12	109091089

First 10 most important SNPs for Asthma's analysis and their corresponding location inside chromosomes.

**Table 3:** Asthma's analysis with Mixed Effect model

Order	SNPs	Gene	**Chrom**.	**Chrom. Pos**.
1	rs2505506	CSGALNACT2	10	43645854
2	rs2813829	no	7	24253168
3	rs739854	no	7	24257317
4	rs7559302	PARD3B	2	205951603
5	rs16218	no	7	24257655
6	rs16212	no	7	24264661
7	rs2642265	no	7	24253062
8	rs16997879	no	20	51909584
9	rs16217	no	7	24257706
10	rs94967	no	21	41195307

First 10 most important SNPs for Asthma's analysis using all samples *n *= 208 with Mixed Effect model.

### Simulation study

In this section we show the results of a small simulation study with the main objective of studying differences among Receiver Operating Characteristic (ROC) curves when considering and ignoring the kinship among individuals. Beside  we consider: the BH method for multiple testing [13,14], the Q-values described in [21], the Efron's procedure in [22] and the ME model also applied for the asthma. It is worth to mention that the comparison of different methods is very difficult and not clear, because each method is specific and tailed for certain desirable features. For instance, among the methods that control an error rate for multiple testing, different methods claim to control the FDR. However, they indeed control different definition of the FDR, because the BH method [13] controls a FDR whose definition differs from the *positive *FDR of the Q-values in [21] and the *local *FDR in [22]. We left to the reader to look at [23] for a general discussion on comparison among these methods. Further more, the three mentioned methods differ from the  in that they do not explicitly estimate the *F*(**x**) model, while they just assume that there exists one that belongs to a certain class of models as the one specified in [14] for the BH procedure. Moreover, the comparison between the importance of a predictor with  and the corresponding significance of a regression coefficient is challenging because they belongs to different metrics as illustrated in [24]. In particular, comparison against the GEE logistic regression discussed in [25] and [26] is left as further interesting work. However, the main advantage of , against regression models, is that nonlinearities and interactions can be learned from the data without any need to be specified beforehand.

Although an extensive comparison of the  with other classification methods is beyond the scope of the paper (on this purposes see, for instance, [27]), we consider a small simulation study were we compare the area under the ROC curve of  and the three mentioned multiple testing approaches with the primary intention of assessing the differences under two scenarios: when the kinship is taken into account, as the case of this paper, and when it is ignored.

Here we simply assume that each method under comparison is able to sort *P *variables according to their level of association, whether or not this is significative. Considering *P *cut points on each list of important variables, we are able to build a ROC curve and measure the corresponding area.

**Figure 4:**
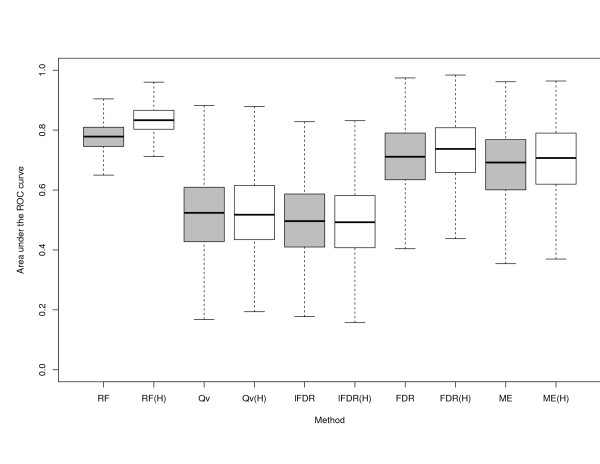
**Results of simulation study**. Each Box-plot represents the sampling distribution of the area under the ROC curve for each considered classification method for 1000 replications. Here RF represents , Qv the Q-values, lFDR the local false discovery rate, FDR the False Discovery Rate and ME the Mixed Effect model. White box-plots indicate samples where cases and controls have been selected using the Hungarian method.

In this simulation study consider *N *= 50 subjects and *P *= 1000 SNPs randomly drawn from the 805 subjects and the 429378 SNPs available from the data set of Thalassemia study where kinship between subjects is known. We assume that only a subset of *p *= 5 SNPs are related to *Y *by a random configuration of the 5 SNPs. This configuration changes for every one of the 1000 draws and cases are assigned to the 10 subjects where Pr(*Y *= 1|*x*_1_, ..., *x_p_*) is the highest. Probabilities are obtained according to the logit transformation of the number of the *p *SNPs that satisfy the random configuration. In this way we always have *I *= 10 cases and 40 controls to be used to form a sample with 2*I *= 20 subjects. Box-plots in Figure 4 illustrate the sample distribution of the area under the ROC curve for the 1000 replications. For each method under comparison we have two sets of box-plots: the grey one where *N *- *I *controls are randomly assigned to cases *I *and the other where they are assigned according to the Hungarian method. Although the limitations of the simulation study, results reflect what found with empirical evidence:  tends to perform better with respect to other methods. It improves when the subjects are selected according to the Hungarian method because of the reduced amount of noise on *P *- *p *non important SNPs. The local false discovery rate and the Q-values perform worse than  and the BH method because they are adaptive methods in the sense that they make an estimation of the null distribution of the *p*-values, while the BH method is not adaptive (for more details see [28]). Estimating the null distribution may be problematic because the sample size is quite small and the observed *p*-values from the Fisher test either concentrate on 0 or on 1. This result validates the choice of the comparison between  and Fisher test made for the application to Thalassemia illustrated in the above section. ME model performs similarly to the BH method. For larger samples, namely *N *> 100, all methods performs equally in terms of ROC curves.

Another message conveyed by Figure 4 is that improvement of methods, under the Hungarian sample, it is not specific to the , but it may be also substantial for other genome-wide scanning methods.

## Conclusions

We propose a strategy analysis that leads to a statistical procedure which allows us to estimate the status of genetic disease, based on the omic profile, and also provides more insights on the biological background of the disease.

Differently from usual methods, we make a multipoint analysis where interactions among all genes are considered and estimated. The method also provides a way to reduce spurious genetic variability by introducing the genealogical information into the analysis through a suitable experimental design.

We think that this strategy may be a reference one for analyzing data from genetic isolated populations, where the degree of relativeness is known.

## Methods

The two critical points of genetic association studies of isolated populations with known genealogy are addressed here. In particular, dependency among subjects is accounted in the following section with a suitable experimental design, based on Hungarian method. The prediction model *F*(**x**) and its estimation, based on Random Forest , is described in the Model Section.

The Hungarian method has been implemented in R (library clue, http://www.r-project.org) while for the  we recurred to the open source project named PArallel Random Forest (PARF, http://code.google.com/p/parf). The latter is implemented in Fortran 95 and compiled with Open MPI libraries http://www.open-mpi.org.

### An experimental design that accounts for genealogy

Estimation of model *F*(**x**), , is based on a subset, **D**, of the available random sample of size *N *, . Variable *y_n _*represents the disease status of the *n*-th subject, where *y_n _*= 1 if the subject *n *has been diagnosed with the disease and *y_n _*= 0 otherwise. Suppose to have  cases and *J *= *N *- *I *controls, where cases and controls have been labeled according to the medical diagnosis of the disease. Vector **x***_n _*is of size *P *and contains SNPs configurations and covariates that resemble habits and individual characteristics of subject n. The vector **x***_n _*defines the omic profile of subject *n *which we use to predict the disease status *Y*.

We are interested in using a balanced sample of size 2*I *where the genetic variability, exogenous to the disease, is reduced. The idea to obtain such sample consists in accounting for relatedness among individuals of different disease status, which we can do taking advantage of the availability of the entire genealogy of the investigated isolated population. For instance, if we consider two individuals, one affected and one not, we are more likely to identify disease predisposing variants if they were brothers rather than if they were unrelated.

The genetic variability, between two individuals, increases with the number of meiotic steps that separate them into the genealogical tree. Such number define relatedness among two individuals. The most common used measure of relatedness between two individuals *i *and *j*, is the *kinship *coefficient [11], *k_ij_*, which represents the probability that two genes, sampled at random from each individual, are identical because inherited from the same ancestor (IBD). For instance *k_ij _*= 1/4 if *i *is the parent of individual *j*, while *k_ij _*= 0 zero if *i *and *j *are not related.

Let  be the matrix of kinship indexes between cases and controls, whose generic entry is *k_ij_*, the kinship index between case *i*, *i *= 1, ..., *I *and control *j*, *j *= 1, ..., *J*. Finding a subset of size I of controls most related to the *I *cases corresponds to find a sub-matrix **K**_*I *× *I *_of **K**' with the *I *columns made by a set of indexes  such that  is maximized over all possible sets of indexes .

This is an assignment problem, which is typical of combinatorial optimization area, and that has been solved with the well known *Hungarian algorithm*. This method was, for many years, attributed to H. Kuhn who developed and published it in [9]. However, in 2006, it was discovered that Carl Gustav Jacobi solved the assignment problem in the 19th century, and published posthumously in 1890 in Latin [10].

In the sequel, we assume that the prediction model *F*(**x**) is estimated on the sub-sample **D **⊂ **D**' where controls correspond to the columns of **K**.

The design of such experiment provides a balanced case/control study with 2*I *individuals, where spurious genetic variability is supposed to have been reduced.

### Model

In order to obtain the prediction model, *F*(**x**), for a certain omic profile, **x**, we propose a procedure based on Random Forest  described in [29].  is a non parametric regression model and its use in genetic association studies grows only in the very recent years. In particular, [27] and [30] use it with expression data, where the considered values of *P *are of order of a few thousands and the observations are assumed independent. On the contrary, most genetic association studies consider only one genetic variant at time and rarely take into account the interactions between them [12]. Other papers, based on multiple testing [23,31], take into account interactions among genes, but they do not explicitly model such dependency as done in this paper.

We briefly describe  with particular emphasis to those variations of the algorithm proposed in this work.  is an additive model given by the ensemble of *M *= 10^6 ^non-parametric classifiers. In our case these are classification trees, , with the usual Bernoulli deviance, where **a***_m _*denotes the regions of the space spanned by the *P *variables. For more details see [32]. Each *h_m _*is based on a random sample of size  of the P variables and a corresponding random sample of size [*I*/2] individuals. This sample is called the *in bag *sample, denoted by **D***_ib _*in order to be distinguished by the out of bag (OOB) sample denoted with **D***_oob _*= **D**\**D***_ib_*. Both sets, **D***_oob_*, **D***_ib _*represent the *m*-th random partition of **D **and the classification error of *h_m _*is always estimated with **D***_oob _*sample of size *I *- [I/2]. This is done in order to avoid overoptimistic prediction errors. The parameter **a***_m _*contains *splitting variables *that are supposed to be the part of the omic profile related to the disease according to the *m*-th tree. The prediction model is given by the mean prediction of the *M *trees:

In this application, each *h_m_*(**a***_m_*) estimates part of the complex relation between the omic profile and the disease, while the whole estimation of such complex model is given by .

If  then the profile **x **belongs to an ill individual otherwise to a healthy one. Note that  is also the Bayes rule under prior probabilities Pr(*Y *= 1) = Pr(*Y *= 0) = 0.5. The threshold, 0.5, may be changed according to different prior probability for the disease of interest. Such prior may be suggested by the prevalence of the disease in the population of interest.

The validation of  is made by means of the prediction error for the outcome *Y *of the *m*-th tree, λ*_m _*(see [29]), estimated over **D***_oob_*. Let Λ*_M _*be the expected prediction error of *M *trees, where expectation is calculated for the joint distribution of (*Y*, **x**). According to Theorems 1.2 and 2.3 in [29], as *M *→ ∞,

where *s *= 1 - *E*_(*Y*,**X**) _(λ*_m_*), is the classifier's strength, and *ρ *is the correlation among classifiers, also known as classifier's diversity. Under conditions of Theorems 1.2 and 2.3,  does not over-fit if we arbitrary increase *M*. A more general discussion on consistency of  and other classification methods can be found in [33].

Using  we obtain a non-parametric regression model that we can use to make prediction and variable (SNP) selection at the same time. Variable selection is performed by means of each *h_m_*, because **a***_m _*is obtained by using only those variables that assure the largest decrease of the Bernoulli deviance.

Therefore, for each of the *P *variables we can consider its *importance *according to the total amount of decreasing of the Bernoulli deviance induced in all *M *trees, say . We can then produce a rank of the *P *variables by sorting them into a decreasing order according to their respective *η_j_*. The interpretation of the set  is that each genetic variant contributes to the complex genetic model, but some of them are more important than others. The greater the *η_j _*the more likely is that variable (SNP) *j *is a risk or protective factor for the disease.

Another advantage of  is that it is highly parallelizable, in fact we implemented it for running in a cluster of CPUs, but it could also be implemented to run on GPUs (Global Processor Units, gpu.sourceforge.net). This approach makes  a suitable approach for association studies with high numbers of combination of genetic variants that interact one with the other. Moreover, the simulation study in [34] showed that  is able to detect true associations under a purely interactive model.

### A Bagging strategy for genome-wide association studies

**Figure 5:**
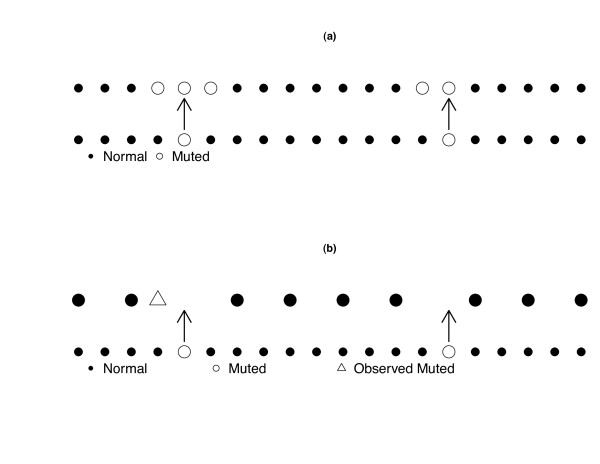
**Clumpy dependence**. (a) Genome configurations if the whole genome were observable: the lower row locates the theoretical mutation points related to the disease, while the upper row locates the mutation points due to Linkage Disequilibrium. (b) Genome configurations when the whole genome is not observed: the lower row locates the theoretical mutation points related to the disease, while the upper row locates the observed points. The triangle corresponds to an observed mutation that able us to trace back the theoretical mutation point.

Alleles of a specific genetic variant are usually associated to the alleles of the neighboring variants due to the lack of recombination between them. This effect is referred to as Linkage Disequilibrium (LD) [35]. LD usually decreases as distance between markers increases. Translating this into statistical terms we have that variables of the omic profile are not independent, but they rather show a clumpy dependence as illustrated in Figure 5(a). Moreover, as the whole genome is not accessible, we exploit LD in order to know the configuration of the variables surrounding the one we have genotyped. This means that, although we do not observe mutations responsible for the disease we can still catch this information through LD as illustrated in Figure 5(b).

Association studies aim at discovering causal variants that are responsible for the disease and these can be located by those SNPs that are nearest to the causal variant. In order to have a good prediction model, we should exclude all SNPs that, although having some predictive power on the outcome *Y*, are far from the causal variant and they are essentially false positives. Statistically speaking, we have to make a screening among all SNPs before proceeding to estimate the final model *F*(**x**). After the screening phase we construct the final model, by selecting the most associated SNPs. These two phases are related to the screening and cleaning procedures advocated in [36]. The work of [36] shows the consistency of this strategy in the context of classical regression models.

For the screening procedure we noted that each *h_m _*makes the subset of the *p *variables randomly chosen to compete, by using, as splitting variables, only those that are most associated with *Y *(i.e. the highest decrease in the Bernoulli deviance). Therefore, only SNPs most related to I are chosen in each tree. If *p *≪ *P *then each **a***_m _*is estimated by the competition between only *p *variables. For this reason it is likely that in *M *trees there would be many different SNPs that are selected to form the final . This approach, which is the usual one in , may produce a final model in which many SNPs are included just because each **a***_m _*refers to different sets of predictors. In order to avoid such false positives, we start by running  in Bagging mode, that is with *p *= *P*, implying that all **D***_ib _*in *M *trees differ only for individuals but not for variables. The set , corresponding to the bagging, allows us to select those SNPs that are most related to *Y *by eliminating all variables with *η_j _*= 0. We actually expect many variables with zero importance, because such variables have never been used in any of the *M *trees. We then repeat the *bagging *procedure on the remaining set of variables until all *η_j _*> 0. This screening phase eliminates all those false positives due only to LD while retaining those SNPs that bear important information. For example, in the case of an association to an haplotype instead of a single SNP on a gene, we expect that only SNPs important to the haplotype would be retained.

We end up, with the screening phase, with a set of *P*' variables and the corresponding model  has a high Λ just because trees only differs for individuals and not for the used variables, therefore its correlation *ρ *→ 1. In order to make *ρ *→ 0, we run again  with the k most important variables among the selected *P*' using the rule suggested in [29], that is  for *k *= 1, ..., P', and  denotes the nearest smallest integer to . We start estimating  with all the elements in the list according to  and we subsequently remove one variable at a time until the estimated value of Λ starts to increase. The final list of variable (SNPs) is the one obtained by running the RF on the increasing sets of most important variables obtained with Bagging. This set is the smallest one that produces the lowest prediction error. This is also the set of variables supposed to be most associated with *Y *and that produces the smallest classification error with .

### Remarks

As final remarks, we would like to comment some aspects of the methods we used:

• all covariates in **X **have almost the same number of categories, 2 or 3, both for SNPs and environmental variables. This is important because it is known that if the number of categories are very different among predictors, or if continuous covariates are mixed with categorical ones, then the latter would result to be most important than categorical variables with a small number of categories. Essentially, there would be a BIAS in assessing variable importance with  as noted in [37]. The BIAS is due to the fact that a variable with more levels or a continuous variable is more likely to be a splitting variable with respect to another discrete variable with a lower number of levels. This could affect situations in which there variables with different number of levels, however it is not the case in our applications. Finally, it would be interesting to compare variable importance by looking at their distance from the root node of the tree as recently proposed in [38] for survival analysis. At the moment, this is beyond the scope of the paper;

• the huge number of predictors, say *P *≈ 500000 (but *P *≈ 10^6 ^is on the wing), requires parallel computing and huge amount of storage and fast access memory. Therefore, it is difficult to manage the kind of analysis, here discussed, by classical regression methods which usually require to calculate (**X***^T^***X**)^-1^. Other alternatives to , based on ensemble methods, could be the gradient boosting algorithm [39] or its stochastic version [40]. From a Bayesian perspective, we note that the Bayesian Additive Regression Trees (BART), proposed in [41], could be an alternative because it allows to draw explicit causal relations among genetic variants and disease status. In our study, we rather draw simple associations. Unfortunately, BART is much more difficult to be parallelize than  and the computational e ort would be not affordable with a standard machine used for the present study;

• the kinship coefficient is a first attempt to summarize the genealogical tree and some information may be lost. To avoid this, it would be possible to directly use the genealogy in the construction of each classification tree *h_m_*. This would be in line with the Breiman's prescription which requires that all aspects of growing a random forest take into account the outcome.

• the proposed design consisting in selecting the controls most inbred with cases could be potentially applied to other methods for association studies, beginning with the very simple Fisher-test as done in the simulation study of Figure 1 for the Thalassemia and also in the simulation study where alternative approaches to the  are compared. At the moment, we have no evidence for stating that the combination of the design and Random Forest has unique advantages;

• rare causal variants, which have a very small effect, can be a problem for RF because each of the *M *trees are grown until the subjects in the final node are less than a specified quantity which is 2 subjects in the present study. Therefore, if there are rare causal variants and they have an effect so small that less than 2 individuals are affected, these cannot be detected with the proposed  approach. However, such variants are, in general, very difficult to be detected.

## Competing interests

The authors declare that they have no competing interests.

## Authors' contributions

SC developed and implemented the statistical method. MEC and FB contributed to the development of the statistical method. NP contributed to development of the method, implemented the method in R and run the RF analysis. LC contributed to the assessment of the obtained results and the construction and management of the data used in both analyses. IP and AS performed the genotyping. GB contributed to the writing. SG clinically supported this research. MP supervised the research. All authors read and approved the final manuscript.
